# Effects of Synbiotic *Lacticaseibacillus paracasei*, *Bifidobacterium breve*, and Prebiotics on the Growth Stimulation of Beneficial Gut Microbiota

**DOI:** 10.3390/foods12203847

**Published:** 2023-10-20

**Authors:** Ekkachai Kaewarsar, Chaiyavat Chaiyasut, Narissara Lailerd, Netnapa Makhamrueang, Sartjin Peerajan, Sasithorn Sirilun

**Affiliations:** 1 Department of Pharmaceutical Sciences, Faculty of Pharmacy, Chiang Mai University, Chiang Mai 50200, Thailand; ekkachai_kaew@cmu.ac.th (E.K.); chaiyavat@gmail.com (C.C.); netnapa.ma@cmu.ac.th (N.M.); 2 Innovation Center for Holistic Health, Nutraceuticals and Cosmeceuticals, Faculty of Pharmacy, Chiang Mai University, Chiang Mai 50200, Thailand; 3 Department of Physiology, Faculty of Medicine, Chiang Mai University, Chiang Mai 50200, Thailand; narissara.lailerd@cmu.ac.th; 4 Office of Research Administration, Chiang Mai University, Chiang Mai 50200, Thailand; 5 Health Innovation Institute, Chiang Mai 50200, Thailand; s.peerajan@gmail.com

**Keywords:** alternative foods, colonic food, food additive, food safety, gut microbiota, prebiotics, probiotics

## Abstract

The gut microbiota is a complex community of microorganisms that plays a vital role in maintaining overall health, and is comprised of *Lactobacillus* and *Bifidobacterium*. The probiotic efficacy and safety of *Lacticaseibacillus paracasei* and *Bifidobacterium breve* for consumption were confirmed by *in vitro* experiments. The survival rate of the probiotics showed a significant decline in *in vitro* gut tract simulation; however, the survival rate was more than 50%. Also, the probiotics could adhere to Caco-2 cell lines by more than 90%, inhibit the pathogenic growths, deconjugate glycocholic acid and taurodeoxycholic acid through activity of bile salt hydrolase (BSH) proteins, and lower cholesterol levels by over 46%. Regarding safety assessment, *L. paracasei* and *B. breve* showed susceptibility to some antibiotics but resistance to vancomycin and were examined as γ-hemolytic strains. Anti-inflammatory properties of *B. breve* with Caco-2 epithelial cell lines showed the significantly highest value (*p* < 0.05) for interleukin-10. Furthermore, probiotics and prebiotics (inulin, fructooligosaccharides, and galactooligosaccharides) comprise synbiotics, which have potential effects on the increased abundance of beneficial microbiota, but do not affect the growth of harmful bacteria in feces samples. Moreover, the highest concentration of short chain fatty acid was of acetic acid, followed by propionic and butyric acid.

## 1. Introduction

Probiotics are described by the International Scientific Association for Probiotics and Prebiotics (ISAPP) as “live microorganisms that, when administered in adequate amounts, confer a health benefit on the host” [1]. They have been reported to possess an advantageous effect on gut health by adjusting the composition and functions of the gut microbiota [2,3]. Among the numerous probiotic strains, *Lactobacillus* spp. (such as *L. paracasei* sp. *paracasei* 19, *L. paracasei* B 21060) and *Bifidobacterium* spp. (such as *B. breve* Bb99, *B. breve* Yakult) have shown promising properties and therapeutic potential [4,5]. Moreover, the yeast *Saccharomyces* spp. (such as *S. cerevisiae* (*boulardii*)) and some *Bacillus* species (such as *B. coagulans* GBI-30, *B. clausii* strains NR, SIN) are also used as probiotics [6]. These microorganisms have been associated with various health benefits, including lactose intolerance improvement, bowel function and gastrointestinal (GI) tract well-being, diarrhea prevention, cholesterol-lowering, hypertension reduction, and immune response regulation [4]. There are multiple strategies available for preserving and improving the efficacy of probiotics, and one particular technique involves the utilization of specific prebiotics. Prebiotics are non-digestible substances that selectively stimulate the growth and activity of beneficial gut microbiota [7]. The enhancement of probiotic functionality by prebiotics is widely recognized in the academic literature. They promote the survival and colonization of specific probiotic strains by acting as substrates for their growth [8].

The combination of prebiotics and probiotics is known as synbiotics. Human fecal fermentation, an *in vitro* method, is commonly used to evaluate the impact of the synbiotics on the growth, diversity, and metabolic activity of gut bacteria [9]. Changes in composition and function in the gut microbiota can also be examined using advanced molecular methods such as next-generation sequencing. The findings may pave the way for innovative synbiotic formulations to promote host–microbiota interactions. The recommendations for food probiotic evaluation define the primary *in vitro* assays for probiotic qualities that must be carried out to evaluate microorganisms for safety and probiotic properties [10]. Effective probiotics should be able to survive gastric acid and bile in the small intestine, adhere to and colonize host gut epithelial cells, and inhibit enteric pathogens [11,12]. Additionally, bile salt hydrolase (BSH) activity is an indicator for probiotic selection [13]. To maintain the balance of the gut microbiota, the BSH hydrolyzes conjugated bile acids into deconjugated bile acids [12].

The human gut microbiota, a complex ecosystem of microorganisms residing in the GI tract, serves a vital role in maintaining overall health and well-being [14]. The composition and activity of the gut microbiota have been associated with a variety of physiological functions, including digestion, immune regulation, and metabolism. Changes in gut microbiota compositions may disrupt gut barrier functions, increase gut permeability, and increase plasma lipopolysaccharide (LPS) concentrations (metabolic endotoxemia), thereby inducing inflammatory bowel disease (IBD), irritable bowel syndrome (IBS), colorectal cancer, allergies, and obesity [15,16]. In recent years, the beneficial interactions of some probiotic strains with various prebiotics to support the development of a beneficial gut microbiota have attracted more attention [17]. Understanding the potential relationships and effects of these synbiotics is necessary for developing focused approaches to improving GI health [17].

This study aimed to verify that *Lacticaseibacillus paracasei* (formerly *Lactobacillus paracasei* [18] and *Bifidobacterium breve* comply with probiotic requirements and are safe for consumption. Additionally, the effects of prebiotics and probiotics as synbiotics on intestinal microbiota and their potential mechanisms (short-chain fatty acids: SCFAs) were investigated.

## 2. Materials and Methods

### 2.1. Bacterial Strains and Culture Medium

*L. paracasei* and *B. breve*, which are included on the probiotic list of the Ministry of Public Health, were manufactured by Lactomason Co., Ltd., located in Gyeongsangnam-do, South Korea. *L. paracasei* and *B. breve* were prepared as freeze-dried powders with final concentrations of 10^11^ CFU/g and 10^10^ CFU/g, respectively. *L. paracasei* (1 g) and *B. breve* (1 g) were inoculated into 10 mL of De Man, Rogosa and Sharpe (MRS) broth (HiMedia Laboratories, Mumbai, India), and MRS broth supplemented with 0.5% *w/v* L-cysteine hydrochloride monohydrate, respectively. The inoculum was then incubated anaerobically at 37 °C for 24 to 48 h.

The pathogenic bacteria, *Bacillus cereus* ATCC 11778, *Escherichia coli* ATCC 25922, *Pseudomonas aeruginosa* ATCC 27853, *Salmonella enterica* subsp. *enterica* ser. Typhi DMST 22842, *Salmonella enterica* subsp. *enterica* ser. Typhimurium TISTR 1469, *Shigella sonnei* ATCC 25931, and *Staphylococcus aureus* ATCC 25923, were obtained from the culture collection of the Faculty of Pharmacy, Chiang Mai University, Thailand. All pathogenic bacteria (10% *v/v*) were cultured in Brain Heart Infusion (BHI) medium (HiMedia Laboratories) and aerobically incubated at 37 °C for 24 h.

Each bacterial suspension was centrifuged for 5 min at 10,000× *g* and 4 °C after incubation. The cell pellet was washed twice with phosphate-buffered saline (PBS) for 5 min at 10,000× *g* and 4 °C. The pellet was then stored at −20 °C in a mixture of culture medium (765 µL) and glycerol (235 µL, 20% *v/v*) until analysis. Before analysis, the bacterial strains (10% *v/v*) were activated and incubated in an appropriate culture medium and under appropriate conditions.

### 2.2. Assessment of Probiotic Properties

#### 2.2.1. *In Vitro* Survival Test for GI Tract Simulation

**Figure 1:**
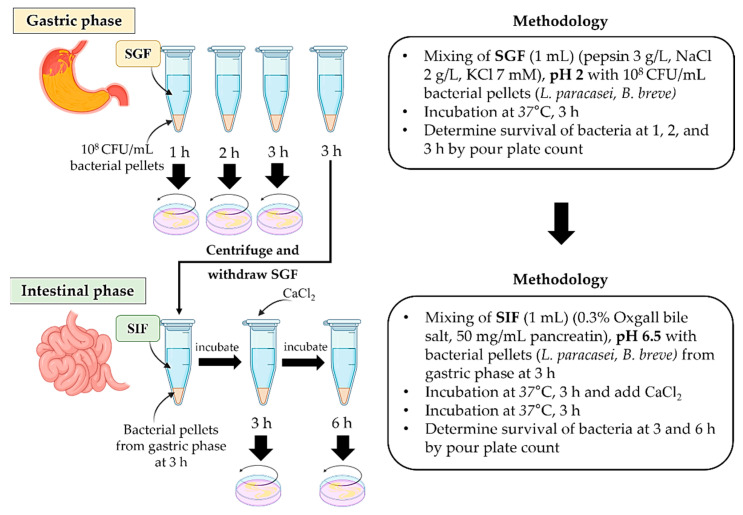
Schematic representation of the *in vitro* survival test for gastrointestinal tract simulation, simulated gastric juice fluid (SGF), and simulated intestinal fluid (SIF).

The purpose of the experiment was to simulate the stomach (simulated gastric juice fluid: SGF) and intestine (simulated intestinal fluid: SIF) mechanisms for the presence of enzymes and pH change according to the methods adapted from García-Ruiz et al. [4], Sadeghi [19], and Campana et al. [20] (Figure 1). The SGF was prepared by collecting 3 g/L of pepsin, 2 g/L of sodium chloride, and 7 mM of potassium chloride, adjusting the pH to 2, and aerobically incubating the mixture for 1 h at 37 °C before use. The SIF was prepared using 0.3% oxgall bile salt and 50 mg/mL of pancreatin, and the pH was adjusted to 6.5.

In the gastric phase, the SGF solution (1 mL) was added to the pellets of probiotic strains and then anaerobically incubated at 37 °C for 3 h. The number of survival bacterial colonies was counted at 1, 2, and 3 h. In the intestinal phase, the pellets from the gastric phase at 3 h were added to the SIF solution and anaerobically incubated at 37 °C for 3 h. After that, the mixture was deactivated using calcium chloride and then continually incubated at 37 °C for 3 h. The number of survival bacterial colonies was counted at 3 and 6 h. The survival of probiotic strains in SGF and SIF was counted by using a pour plate technique. Briefly, an aliquot of the mixture (1 mL) was centrifuged at 7000× *g* at 4 °C for 10 min and rinsed twice with PBS buffer (pH 7). The pellet was diluted by a 10-fold PBS buffer dilution. The dilution (1 mL) was poured in molten MRS agar supplemented with 0.2% *w/v* bromocresol purple and let to stand at room temperature until solidified. The plate was anaerobically incubated at 37 °C for 24–48 h. The survival rate of the bacterial number (log CFU/mL) was calculated as the percentage using the equation below (1).

Survival rate of bacteria (%) = (N_1_/N_2_) × 100 (1)

where N_1_ is the number of bacterial colonies (log CFU/mL) at the end of incubation time, while N_2_ is the number of bacterial colonies (log CFU/mL) at the initial time.

#### 2.2.2. Adhesion Ability to Caco-2 Cell Lines

The adherence ability of probiotic strains was examined using colorectal adenocarcinoma Caco-2 cell lines (No. RCB0988, RIKEN BioResource Research Center, Ibaraki, Japan) according to the adapted method of Pennacchia et al. [21], García-Ruiz et al. [4], and Panicker et al. [12]. The cells were cultured in Dulbecco’s modified Eagle’s medium (DMEM) (Gibco, Grand Island, NY, USA) supplemented with 10% fetal bovine serum (FBS) (Gibco), 2 mmol/L of L-glutamine (Gibco), 100 U/mL penicillin (Gibco), and 30 µg/mL streptomycin (Gibco) at 37 °C containing 5% carbon dioxide and with 100% relative humidity. A 5 mL quantity of Caco-2 cells, approximately 10^5^ cells/mL, was transferred to a six-well tissue culture plate and cultured until 80 to 85% confluence.

The adherence activity was adapted from the methods of Panicker et al. [12], Pennacchia et al. [21], and García-Ruiz et al. [4]. The probiotic pellet was resuspended in the PBS buffer with a final concentration of 10^8^ CFU/mL. The suspension was centrifuged for 5 min at 10,000× *g*, 4 °C, and resuspended in 4 mL non-supplemented DMEM media (pH 8). The suspension was added to a Caco-2 cell monolayer-containing well and incubated at 37 °C with 5% CO_2_ for 2 h. After that, the samples were rinsed five times with PBS buffer. The samples were added to a 0.25% trypsin–EDTA solution and left at room temperature for 10 to 15 min. The bacterial number was determined by plating (spread plate) serial 10-fold dilutions of the mixture with PBS buffer and cultivating on MRS agar for 24–48 h at 37 °C. The percentage of adhesion was determined using Equation (2), below.

Adhesion range (%) = (A/B) × 100(2)

where A represents the amount of bacterial adhesion (CFU/mL) at the end of the test and B represents the amount of bacteria (CFU/mL) at the initial time.

#### 2.2.3. Antimicrobial Activity

The antimicrobial activity of the probiotic strains was determined using the agar spot [11] and agar well diffusion tests [19,20,22]. Seven pathogenic bacteria were used as indicators, including *B. cereus*, *E. coli*, *P. aeruginosa*, *S.* Typhi, *S.* Typhimurium, *S. sonnei*, and *S. aureus*. For the agar spot test, the probiotics (10^8^ CFU/mL) were spotted on the BHI medium and anaerobically incubated at 37 °C for 24 h. Each pathogenic strain (10^6^ CFU/mL) was overlaid on the BHI medium and aerobically incubated at 37 °C for 24 to 48 h. The inhibition zones around the colony spot were measured by their diameter, not including the diameter of the colony spot. In the case of the agar well diffusion test, the pathogenic bacteria were suspended in BHI medium agar and added to the culture plates. A 6 mm diameter well was made on agar plates using a cock borer. The supernatants of probiotic cultures were filtered with a 0.22 µm filter membrane (CNW Technologies, Shanghai, China) and then applied to each slot. The plates were incubated at 37 °C for 24 h. The diameters of the inhibition zones surrounding the slot were measured, excluding the diameter of the slot.

#### 2.2.4. Bile Salt Hydrolase (BSH) Activity

The BSH activity of probiotic strains was based on the method described by Ayama et al. [11], Kumar et al. [23], and Pereira et al. [24]. The suspensions of probiotics were spotted on MRS agar plates supplemented with 0.5% ***w/v*** conjugated bile salt (glycocholic acid (GCA), taurocholic acid (TCA), and taurodeoxycholic acid (TDCA) from Fluka, Sigma-Aldrich, St. Louis, MO, USA) and 0.37 g/L calcium chloride. The MRS agar plate without supplementation with bile salts was used as a control. The plates were anaerobically incubated at 37 °C for 24, 48, and 72 h. BSH activity was determined through precipitation zones around the colonies.

#### 2.2.5. *In Vitro* Evaluation of Cholesterol Lowering

Changes in the cholesterol concentrations of MRS broth after the culturing of the probiotic strains were determined using high-performance liquid chromatography (HPLC) [23,25,26,27]. The probiotics were cultured with the concentration adjusted to 10^8^ CFU/mL in MRS broth supplemented with 5 mg/mL of water-soluble cholesterol (cholesterol-PEG 600, Fluka, Sigma-Aldrich, St. Louis, MO, USA) and anaerobically incubated at 37 °C for 48 h. The samples (at 0 and 48 h) were centrifuged at 10,000× *g* at 4 °C for 5 min. The supernatant (1 mL) was mixed with 3 mL of 95% ethanol and 2 mL of 50% potassium hydroxide. The mixture was heated at 60 °C for 10 min and cooled to room temperature. Then, 5 mL of hexane and 3 mL of distilled water were sequentially added and left for 15 min at room temperature until phase separation. An aliquot of 2.5 mL of the hexane layer was evaporated at 60 °C under the flow of nitrogen gas. The residual cholesterol was suspended in 1 mL of acetonitrile and isopropanol (in a ratio of 95:5) and filtered with a 0.22 µm filter membrane for the residual cholesterol analysis using HPLC (model LC-20AD, Shimadzu, Kyoto, Japan) with a 120 EC-C18 column (4 µm, 4.6 × 150 mm, Poroshell, Agilent Technologies, Santa Clara, CA, USA). The mobile phases were a mixture of acetonitrile (95%) and isopropanol (5%), the flow rate was 2 mL/min in isocratic elution, and the samples were detected at 202 nm wavelength. The amounts of cholesterol were calculated and reported as a percentage of cholesterol lowering using Equation (3), below.

Cholesterol lowering (%) = [(A − B)/A] × 100 (3)

where A is the cholesterol concentration at the initial time (0 h) and B is the cholesterol concentration at the collection time.

### 2.3. Safety Assessments of Probiotics

#### 2.3.1. Antibiotics Susceptibility

Antibiotics susceptibility was determined by the agar disc diffusion method using antibiotic discs following the guidance on the assessment of bacterial susceptibility to antimicrobials of human and veterinary importance, European Food Safety Authority (EFSA) [28]. Antibiotic discs included ampicillin (10 µg/disc), chloramphenicol (30 µg/disc), clindamycin (2 µg/disc), erythromycin (15 µg/disc), gentamycin (10 µg/disc), kanamycin (30 µg/disc), tetracycline (30 µg/disc), streptomycin (10 µg/disc), and vancomycin (30 µg/disc). The probiotics were cultured in MRS broth at anaerobe conditions, 37 °C for 24 to 48 h. The bacterial suspension was centrifuged at 10,000× *g*, 4 °C for 5 min. The bacterial pellets were harvested and resuspended with PBS buffer (1 mL). The solution was adjusted to 10^7^ CFU/mL by using a spectrophotometer at 570 nm at OD 0.6. The bacterial suspension was swabbed on an MRS agar plate and dried under UV light for 15 min in a biosafety cabinet. The antibiotic discs were placed onto the plates and anaerobically incubated at 37 °C for 24 h. The diameter of the inhibition zone was measured using vernier calipers. The zone diameters were compared with the standard Zone Diameter Imperative Chart that was supplied with the antibiotic disc (adapted from NCCL standards by BBL). The susceptibility was expressed in terms of resistance (R), intermediate (I), and susceptible (S).

#### 2.3.2. Hemolytic Activity

The probiotic culture was streaked on tryptone soya agar (TSA) culture medium (HiMedia Laboratories) supplemented with sterile sheep blood (7% *v/v*) (Envimed Co. LTD., Bangkok, Thailand) and then anaerobically incubated at 37 °C for 24 to 72 h. After incubating, the blood agar plates were examined for hemolytic activity. Only strains with γ-hemolytic are considered as safe [29].

### 2.4. Anti-Inflammatory Properties of Probiotics in Caco-2 Epithelial Cell Lines

The anti-inflammatory assay involved a modified method from De Marco et al. [30] and Aghamohammad et al. [31]. Briefly, Caco-2 cell lines were cultured in a manner similar to Section 2.2.2. The cells were transferred to a 24-well tissue culture plate and cultured until 80 to 85% confluence. The cells were co-cultured with 100 ng/mL LPS (Sigma-Aldrich, Ottawa, ON, Canada), *E. coli, S.* Typhi, *L. paracasei*, *B. breve*, LPS + *L. paracasei*, LPS + *B. breve, E. coli* + *L. paracasei, E. coli* + *B. breve, S.* Typhi + *L. paracasei*, and *S.* Typhi + *B. breve*. The culture plates were anaerobically incubated at 37 °C for 18 h in 5% CO_2_. After that, the samples were centrifuged at 6000× *g* for 10 min and the supernatants were collected for cytokine analysis. The concentration of secreted cytokines and chemokines in cell-free supernatants (CFS), including interleukin (IL)-6, IL-10, IL-12, was evaluated using Quantikine^®^ Colorimetric Sandwich ELISA Kits (Quantikine by R&D Systems, Minneapolis, MN, USA) according to the manufacturer’s instructions.

### 2.5. Synbiotic Powder Preparation

The probiotic powders, namely *L. paracasei* and *B. breve,* were present at a final concentration of 10^9^ CFU/g. The prebiotic ratios of inulin (INU), fructooligosaccharides (FOS), and galactooligosaccharides (GOS) were 1.33, 2.00, and 2.67% *w/v*, respectively [32]. The synbiotic formulation consisted of a 1:1 combination of probiotics and prebiotics. The products were contained in a foil sachet before the experiment. The stability of synbiotic powder at 5 and 30 °C for 6 months focused on the survival of probiotics, moisture content, and water solubility index.

### 2.6. In Vitro Test of Gut Microbiota Stimulation in Feces

The feces samples were obtained from healthy volunteers in the previous study. The study was reviewed and approved by the Thai Clinical Trials Registry (TCTR) Committee (TCTR identification number is TCTR20200923003; https://www.thaiclinicaltrials.org/show/TCTR20200923003 (accessed on 18 September 2023)). One gram of feces sample was suspended in a solution of modified culture medium (10 mL), Tween 80 (2 mL), phylloquinone (10 μL), and hemin solution (1 mL) [33,34,35]. The pH of the solution was adjusted to 6.5 with sodium hydroxide (1 M). The synbiotic powder (1 g) was mixed into the solution, followed by anaerobic incubation for 48 h at 37 °C. The specimens were obtained at time intervals of 0, 12, 24, and 48 h and transferred to a micro-centrifuge tube (1.5 mL). The tubes were centrifuged at 10,000× *g* for 20 min. The fecal sample pellets were analyzed for the abundance of fecal gut microbiota by using real-time quantitative PCR while the supernatant was analyzed for organic acid contents by using HPLC. 

### 2.7. Quantification of Gut Microbiota in Feces Using Real-Time Quantitative PCR

Microbial DNA in the feces sample was extracted using a commercial kit (NucleoSpin^®^ DNA feces, Dueren, Germany) according to the manufacturer’s instructions. Real-time PCR was performed with the QuantStudio™ 6 Flex Real-Time PCR System in a 96-well Fast reaction plate (Applied Biosystems™, Waltham, MA, USA). The quantitative real-time PCR (qPCR) based on SYBR Green was used to characterize the gut microbiota by using specific primers targeting 16S rRNA genes of different target bacterial groups [36]. The specific primers of *Bifidobacterium* spp., *Lactobacillus* spp., Bacteroidetes, Firmicutes, *Escherichia coli*, and total bacteria are shown in Table 1. Each reaction was performed using 2 μL of DNA template and 18 μL of the PCR mixture. The PCR mixture comprised 10 μL of SYBR (Fast SYBR™ Green Master Mix, Applied Biosystems™, Waltham, MA, USA), 0.2 μL of each primer, and 7.6 μL of distilled water. The real-time PCR condition was as follows: 95 °C for 2 min, followed by 45 cycles of 95 °C for 20 s and 60 °C for 20 s. The melting curve was obtained by performing the following cycles: 95 °C for 20 s, 60 °C for 20 s, and 95 °C for 20 s. The quantification of gut microbiota was calculated with the standard curves of bacterial reference strains. The standard curves were prepared using 10^2^–10^10^ CFU/mL of the 16S rRNA genes of bacterial reference strains [37].

### 2.8. Determination of Organic Acids

The content of lactic acid, acetic acid, propionic acid, and butyric acid was determined using the HPLC technique [19,22,23,24]. The supernatant of the feces samples (500 μL) was well mixed with 500 μL of 5 mM sulfuric acid (RCI Lab-scan, Bangkok, Thailand). The solution was filtered through a 0.22 μm filter and stored in an amber glass vial tube. A SUGAR column (6 µm, 8 × 300 mm, SH1011, Shodex, Munich, Germany) was used to identify these organic acids. The analytical column was maintained at a constant temperature of 75 °C. The sample solution was eluted with 5 mM sulfuric acid, which was used as a mobile phase. The flow rate was 0.6 mL/min, and a UV detector set to 220 nm detected the organic acids.

### 2.9. Stability Assessments of Synbiotics

The synbiotics were contained in a foil sachet and stored at 5 ± 3 and 30 ± 2 °C for 6 months according to the guidelines [43]. The humidity of synbiotics at 0, 1, 3, and 6 months was measured using a moisture analyzer (Sartorius, MA50, Wood Dale, IL, USA). The survival probiotics were cultured on MRS with bromocresol purple and anaerobically incubated at 37 °C for 24–48 h. The survival rate was determined in the survival bacterial colonies grown on a selective culture medium. For the water solubility of synbiotics, distilled water (10 mL) was added to the synbiotics (1 g) and mixed every 5 min for 30 min. Then, the mixture solution was centrifuged at 5000× *g* for 10 min. The supernatant was transferred to a moisture can and incubated at 100 °C for 8 h or until dried. The water solubility index was calculated using Equation (4), below [44].

Water solubility index (%) = (A/B) × 100(4)

where A is the dry solid weight (g), while B is the initial sample weight (g).

### 2.10. Statistical Analysis

All results of each experiment were determined in triplicate and expressed as mean values with standard deviations (SD). ANOVA and post hoc Tukey HSD multiple comparisons among means were performed using statistical SPSS Software (Version 17, SPSS Inc., Chicago, IL, USA) to analyze the significant differences in the anti-inflammatory properties of probiotic supernatants in Caco-2 epithelial cell lines, the quantification of fecal microbiota, and organic acid contents. The paired *t*-test was performed to analyze the significant differences in cholesterol-lowering, and a synbiotics stability test was carried out. A *p* < 0.05 was considered statistically significant.

## 3. Results and Discussion

### 3.1. Probiotic Properties

#### 3.1.1. *In Vitro* Survival Rate of Probiotics in GI Tract Simulation

The viability of probiotics within the GI system is a crucial characteristic of their role as probiotics and their beneficial impact on the host. To be effective, probiotics should possess resistance to the acidic conditions of the stomach and the presence of bile salts in the small intestine [11,19]. The differential survival rates of different probiotic strains in SGF and SFI can be attributed to various factors related to the characteristics of the strains themselves and the conditions of the simulated environment.

**Figure 2:**
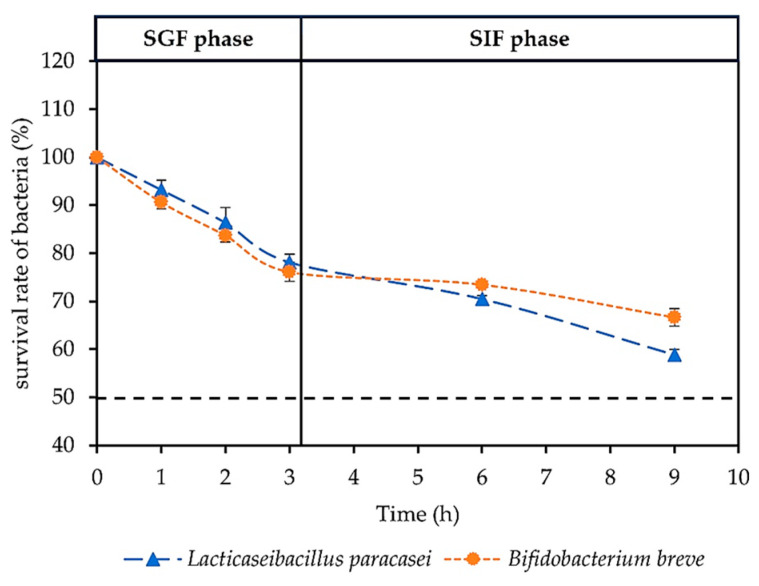
The survival rate at percentage of log number of of *Lacticaseibacillus paracasei* and *Bifidobacterium breve* in the simulated gastric juice fluid (SGF) phase and simulated intestinal fluid (SIF) phase.

The present investigation observed that the survival rate of the probiotic strains, namely *L. paracasei* and *B. breve*, showed a significant decline as time progressed. In the case of SGF, *L. paracasei* had a percentage of survival rate of about 93.22 ± 1.96, 86.38 ± 3.15, and 78.13 ± 1.63% at 1, 2 and 3 h, while *B. breve* exhibited survival rates of 90.62 ± 1.45, 83.69 ± 1.39, and 76.07 ± 1.97% at 1, 2, and 3 h, respectively. The study on the ability to survive of probiotics in SIF with a pH of 6.5 revealed that *L. paracasei* exhibited survival rates of approximately 70.50 ± 0.68% and 58.87 ± 1.07% after 3 and 6 h of the SIF phase, respectively. Similarly, *B. breve* demonstrated a survival rate of approximately 73.43 ± 0.58% and 66.61 ± 1.84% after 3 and 6 h of SIF phase, respectively. However, the probiotics exhibited a survival rate over 50% when exposed to SGF and SIF, as shown in Figure 2.

LAB strains, which are classified as probiotics, exhibited a greater than 50% survival rate when exposed to low pH gastric juice conditions and a bile salt concentration of 0.3% for 4 h, according to a literature review. In brief, the *Lactobacillus brevis* MH748630 exhibited the maximum level of bacterial survival (80.84%) in low pH conditions, whereas the *Leuconostoc lactis* MH748629 showed the highest survival rate (83.17%) in the presence of bile [45].

After passing through the mouth and esophagus, when the probiotics enter the stomach, they must contend with the low pH of the gastric fluid [46]. The stomach gastric fluid contains pepsin and lipase enzymes for protein and lipid digestion, while hydrochloric acid (HCl) results in a decreased gastric pH from 6 to 1.5 for protein hydrolysis [47] and to eliminate invasive bacteria [46]. Once activated, pepsin can interact with the peptide component of the cell wall, while lipase can destroy phospholipids and glycolipids in cell membranes of microorganisms [46]. In addition, acidity possesses antibacterial properties, disrupts enzymatic processes, damages DNA, and acidifies the cytoplasm, which impacts membrane potential [46]. Quite possibly, the probiotic strains naturally possess mechanisms providing enhanced resistance to acidic conditions and other stresses in the stomach. These mechanisms could involve proton pumps, the adaptation of the hydrophobicity of the cell surface, the maintenance or repair of cell components, and metabolic changes, all contributing to maintaining the intracellular pH in low-pH environments [48], resulting in higher survival rates. In addition to acidic conditions, the presence of bile salts in the small intestine might also present a challenge for probiotics. The passage through the stomach takes 15 m to 3 h, and then the probiotic bacteria reach the intestines [46,47]. The intestines contain pancreatic enzymes, proteases, amylases, lipases, and bile, acting together to break down food constituents [47,49]. Additionally, bile possesses antibacterial effects at concentrations greater than 40 mM, regulating the microbiota in the intestines. Thus, probiotics must be able to resist many enzymes and bile acids [46]. The mechanisms that maintain probiotic viability at an alkaline pH demonstrate the expression of genes that code for the so-called alkaline shock proteins (Asp), enabling their survival in the sudden pH change from acidic to alkaline [50]. Other mechanisms include (i) active potassium extrusion and the potassium–proton antiport system, (ii) the sodium–proton antiport system, (iii) proton-translocating adenosine triphosphatase (ATPase), (iv) the formation of transmembrane proton gradients (ΔpH) in a reversed direction, and (v) cross-protection and changes in protein synthesis [51]. The viability of probiotics can be affected by the presence of food or other substances within the digestive system. Some components may exhibit a protective effect or engage in interactions with the gastric ecosystem contributing to sustaining probiotic viability. In some cases, the viability of probiotics may be augmented by combining with prebiotics, which are compounds promoting the growth of beneficial microbes, or incorporating in synbiotic formulations. The combination could lead to the development of increasingly suitable conditions for the survival and growth of probiotic microorganisms.

#### 3.1.2. Adhesion Ability of Probiotics to Caco-2 Cell Lines

The adhesion ability of probiotics in the gastric ecosystem refers to the capacity of the microorganisms to colonize the GI tract and exert their beneficial effects. Some strains have evolved capacities that attach to the gut epithelium better than others. The current study observed a decrease in the adherent population of *L. paracasei*, with a mean value of 7.37 ± 0.10 log CFU/mL compared with the starting population of 8.03 log CFU/mL. The adhesion to epithelial cells of *L. paracasei* was determined to be 91.79 ± 1.29%. *B. breve* exhibited a high level of adhesion, with an approximate value of 7.50 ± 0.08 log CFU/mL of a total of 8.03 log CFU/mL supplied. The adherence ability of *B. breve* was estimated to be about 93.38 ± 0.96%. In the related investigation mentioned, Tham et al. [52] found that *L. casei* BT 1268 and *B. longum* FTDC 8643 exhibited adherence rates of around 78.89 ± 0.84% and 69.25 ± 1.65%, respectively. Adhesive probiotic strains can connect to these mucosal cells through molecules or structures on their surfaces and gut cells. This colonization protects them from hazardous microorganisms and creates a competitive environment, limiting pathogen development [4]. In addition, the interactions with host cells alter immune responses, gut barrier function, and signaling molecule release, affecting digestion and other physiological functions [52]. The adhesion of *Lactobacillus* and *Bifidobacterium* to the mucosa is the first step in binding by non-specific hydrophobics, followed by a second step using specific cell wall components and mucin-binding proteins [53]. However, the adherence on the Caco-2 cell lines has limitations because of an absence of mucus and motility.

#### 3.1.3. Antimicrobial Activity

Antimicrobial activity is one of the ways in which probiotics exhibit their beneficial properties. The range of pathogens is important to consider as an indicator for antimicrobial tests in probiotic research. The present study is interested in antimicrobial activity against GI pathogens. These can include bacteria such as *B. cereus*, *E. coli*, *P. aeruginosa*, *S*. Typhi, *S*. Typhimurium, *S. sonnei*, and *S. aureus*, which are known to cause various digestive disorders and infections. *L. paracasei* and *B. breve* displayed all pathogenic antibacterial activity, as shown in Table 2. In the agar spot test, *L. paracasei* exhibited strong inhibitory effects on *E. coli*, *P. aeruginosa*, *S.* Typhi, and *S. aureus*, while displaying moderate inhibitory effects on *B. cereus, S.* Typhimurium, and *S. sonnei*. Conversely, *B. breve* demonstrated strong inhibitory effects on *S. aureus*, moderate inhibitory effects on *B. cereus, E. coli*, *S.* Typhi, *S.* Typhimurium, and *S. sonnei*, and weak inhibitory effects on *P. aeruginosa*. In the agar well diffusion, *L. paracasei* inhibited *S*. Typhi and *S. sonnei* strongly, *E. coli*, *P. aeruginosa*, *S.* Typhimurium, and *S. aureus* moderately, and *B. cereus* weakly, whereas *B. breve* inhibited *E. coli* and *S. aureus* strongly, *B. cereus*, *P. aeruginosa*, *S*. Typhi, and *S. sonnei* moderately, and *S.* Typhimurium weakly. The results of the agar spot test and agar well diffusion experiments demonstrated the significant antibacterial activity of *L. paracasei* against *S*. Typhi, but *B. breve* exhibited high inhibition against *S. aureus*. Not all probiotics possess the same antimicrobial properties, and their efficacy can be affected by variables such as intestinal microbiota composition, nutrition, and host.

In a study by Gheziel et al. [54], all strains of *L. plantarum* showed significant antibacterial activity against *E. coli* O157:H7 CECT 4267 and all tested strains of *S. aureus*, as shown by inhibition zones of more than 6 mm. In another study, *L. plantarum* RYPC5 isolates exhibited the maximum inhibition zone against *E. coli*, followed by good inhibition zones against *S. aureus*, *P. aeruginosa*, and *S. albony*, while *L. plantarum* RYPR1 also showed good inhibition zones against *E. coli*, *S. aureus*, *P. aeruginosa*, and *S. albony* [55].

Probiotics can exhibit antibacterial characteristics through several mechanisms: (i) probiotic bacteria can engage in competitive interactions with pathogenic microbes for space and resources, thereby limiting the growth of harmful bacteria and contributing to the maintenance of a balanced intestinal microbiome; (ii) probiotics can synthesize bioactive compounds including organic acids (such as lactic acid), hydrogen peroxide, bacteriocins (protein molecules that are toxic to other bacteria), and other metabolites [56]. These compounds can produce conditions inhibiting the growth of pathogens. The gut barrier serves as a defensive barrier, effectively inhibiting the translocation of pathogenic bacteria and their associated metabolites into the systemic circulation, reducing the risk of infection [57].

#### 3.1.4. Bile Salt Hydrolase (BSH) Activity

BSH is the enzyme responsible for catalyzing the hydrolysis of glycine- or taurine-conjugated bile acids into the deconjugated bile acids (free bile acids) and amino acids, where BSH enzyme is commonly found in *Lactobacillus* and *Bifidobacterium* species [58,59]. *L. paracasei* and *B. breve* had BSH activity only at 48 and 72 h of incubation time, which showed a white precipitate around the colonies. *L. paracasei* and *B. breve* showed positive BSH activity on MRS supplemented with GCA and TDCA, while not showing BSH activity on MRS supplemented with TCA (Table 3).

Similarly, Kumar et al. [23] reported and discussed how 12 lactobacilli showed positive GCA- and TDCA-hydrolase activity but showed negative activity for taurocholic acid (TCA) hydrolysis. These results indicate that the substrate specificity of BSH enzymes may be influenced either by the amino acid moiety (glycine or taurine) in the conjugate or by other side chains on the steroid moiety. In total, 12 strains of *Bifidobacterium* exhibited a distinctive activity of BSH. Specifically, *B. catenulatum*, *B. longum* subsp. *longum*, and *B. longum* subsp. *suis* were shown to create colonies with an opaque white appearance [60]. The BSH activity of probiotics in the intestine could modify the conjugated bile acids into deconjugated bile acids that were less soluble and absorbed by the intestines, leading to their elimination in the feces, which would increase the *de novo* synthesis of conjugated bile acids from cholesterol to maintain the hepatic bile acid pool [61] and replace bile salts lost with excretion [26], or reduce cholesterol solubility and its subsequent absorption by the intestinal lumen [46], leading to reduced serum cholesterol [26].

#### 3.1.5. Cholesterol Lowering

Probiotics have been reported to possess an important impact on reducing cholesterol levels [62] and triglycerides, and preventing and treating diabetes and obesity [46]. The findings of the study on cholesterol reduction revealed that *L. paracasei* and *B. breve* could reduce cholesterol levels in a culture medium. *L. paracasei* exhibited reduced cholesterol levels in a culture medium of around 46.33 ± 0.70% over the 48 h incubation period. Similarly, *B. breve* demonstrated a percentage change in cholesterol levels of approximately 46.88 ± 0.23% during the 48 h incubation period. 

A related study reported that a total of 85 strains of LAB could reduce cholesterol levels in a medium broth, with a range of effectiveness ranging from 3.8% to 55.2% [63]. *L. plantarum* ATCC 14917 had the highest ability to assimilate cholesterol in MRS broth, while *L. reuteri* NCIMB 701089 demonstrated a cholesterol assimilation rate of 67% [27]. Additionally, *B. bifidum* MB107 and *B. bifidum* MB109 could remove 81 and 50 mg of cholesterol per gram of biomass, respectively [26].

Many mechanisms have been proposed for cholesterol-lowering properties via probiotics, such as the deconjugation of primary bile salts into secondary bile salts by the BSH enzyme, the production of SCFAs (acetate, propionate, and butyrate) during growth, the assimilation of cholesterol into the cell membrane, and/or the conversion of cholesterol to coprostanol [24,46]. Moreover, probiotics can co-precipitate cholesterol with deconjugated bile salts in an acidic environment, contributing to cholesterol removal [27,58].

### 3.2. Safety of Probiotics

#### 3.2.1. Antibiotic-Resistant Probiotics

Understanding the susceptibility of probiotics to antibiotics helps ensure that the strains employed in probiotic products contain no antibiotic-resistance genes that may potentially transfer to pathogenic bacteria within the gut tract [64]. The use of probiotics that are resistant to antibiotics has the potential to promote the development and spread of antibiotic-resistance. The antibiotic susceptibility of probiotics is shown in Table 4. *L. paracasei* has been shown to exhibit susceptibility to six antibiotics, namely ampicillin, erythromycin, gentamycin, tetracycline, chloramphenicol, and streptomycin, whereas it has demonstrated complete resistance to vancomycin, kanamycin, and clindamycin. *B. breve* exhibited sensitivity to ampicillin, erythromycin, gentamycin, tetracycline, and streptomycin, while indicating intermediate susceptibility to chloramphenicol, kanamycin, and clindamycin. Furthermore, *B. breve* exhibited complete resistance to vancomycin.

Previously, Yadav et al. [55] reported that all probiotics in the LAB showed resistance to vancomycin, and some isolates showed resistance to kanamycin. Resistance to kanamycin is due to a lack of cytochrome-mediated electron transport in LAB, which mediates the antibiotics’ absorption [65]. Duche et al. [66] found that *L. paracasei* with *ermB* and/or *ermC* was sensitive to both macrolide antibiotics. The *ermB* and/or *ermC* genotype (plasmid, transposon, and chromosome locations) are also linked to phenotypic resistance to the lincosamide clindamycin [67]. Halder et al. [68] and Plessas et al. [69] revealed that all the isolates of *Lactobacillus* and *L. paracasei* K5 were resistant to vancomycin, which constitutes a cell wall synthesis inhibitor [68,69]. They also described how this resistance is considered a natural or intrinsic property, which is due to the presence of D-alanine: D-alanine ligase-related enzymes are used to separate them from other Gram-positive bacteria. The best-characterized resistance in LAB is its resistance to vancomycin, due to the lack of binding sites in peptidoglycan [65].

Most research has shown that *Lactobacillus* species are typically sensitive to several antibiotic medication classes, such as penicillin, chloramphenicol, tetracycline, quinupristin-dalfopristin, macrolide (erythromycin), lincosamide (clindamycin), oxazolidinone (linezolid), and rifampin. Antibiotic-resistance in this category of bacteria is classified as intrinsic and acquired due to the fact that these microorganisms are inherently resistant to a number of antibiotics. On the other hand, chromosomal gene mutation or horizontal gene transfer (HGT) results in acquired resistance. Intrinsic and chromosomally altered genes are unlikely to be transmitted to other bacteria since HGT is the primary method of resistance transmission [70].

#### 3.2.2. Hemolytic Activity

The assessment of hemolytic activity is considered a safety criterion in selecting probiotic strains [71]. The process of hemolysis can result in the release of hemoglobin and various cellular constituents in the blood vessels, which may give rise to negative effects such as tissue damage and inflammation. Testing the hemolytic activity of probiotics ensures that they do not pose a risk of damaging the host or injuring red blood cells. Additionally, the selection of probiotics must consider a lack of hemolytic activity because such strains are non-virulent and the lack of hemolysin ensures that virulence will not appear among the bacterial strains [29]. The production of an enzyme that can break down mucin was proposed as a determinant factor of virulence for some pathogens. This property leads to alterations in the intestinal mucosal barrier and intrusion by pathogens and toxic agents [72]. In the present study, the hemolytic activity of *L. paracasei* and *B. breve* showed no change around colonies, indicating a lack of hemolytic activity. These strains were identified as γ-hemolytic strains, suggesting their suitability for use as probiotics. According to Halder et al. [68], the isolated lactobacilli did not exhibit any distinct transparent or greenish zone surrounding their colonies on the blood agar plates. As a result, the lactobacilli were classified as either γ-hemolytic or non-hemolytic.

### 3.3. Anti-Inflammatory Properties of Probiotic Supernatants in Caco-2 Epithelial Cell Lines

Probiotics play important roles in improving health, such as modulating the gut microbiota, producing activities against pathogens by regulating mucus secretion, and regulating the immune system, showing anti-inflammatory effects [31]. LPS, which is generated from the cell wall of Gram-negative bacteria like *E. coli* and *Salmonella*, is the main catalyst for intestinal epithelial cell inflammation and important immunomodulatory components [73]. Several probiotic strains can stimulate dendritic cells, which then transfer antigens to nearby lymph nodes and produce IL-10, IL-12, IL-1β, and IL-6 [74]. IL-10 is an anti-inflammatory cytokine inhibiting the proinflammatory cascade, while IL-6 and IL-12 are proinflammatory cytokines [30].

In the present study, the secretion of cytokines by Caco-2, including IL-6, IL-10, and IL-12, showed the significantly highest value (*p* < 0.05) when treated with LPS for IL-6 (55.15 ± 1.98 pg/mL), IL-12 (7.56 ± 0.27 pg/mL), and *B. breve* for IL-10 (283.53 ± 4.10 pg/mL) (Table 5). In addition, all treatments showed a significant (*p* < 0.05) induced secretion of IL-6, higher than untreated (5.85 ± 0.38 pg/mL). *L. paracasei* showed the significantly lowest (*p* < 0.05) induced IL-6 (17.85 ± 1.85 pg/mL) when compared with other treatments, except untreated. However, *B. breve* showed the significantly lowest (*p* < 0.05) induced IL-12 (2.39 ± 0.21 pg/mL) when compared with other treatments, except untreated. LPS, *E. coli*, *S.* Typhi, and the co-cultured treatment showed a significant (*p* < 0.05) induced secretion of IL-6 and IL-12, higher than only the probiotics. Interestingly, the secretion of the anti-inflammatory cytokine IL-10 showed the significantly lowest values (*p* < 0.05) when treated with LPS (83.93 ± 0.89 pg/mL) but showed the significantly highest ones (*p* < 0.05) when treated with probiotics, *B. breve* (283.53 ± 4.10 pg/mL) and *L. paracasei* (264.65 ± 0.82 pg/mL), followed by co-cultured LPS with *B. breve* (221.56 ± 3.09 pg/mL) and LPS with *L. paracasei* (216.32 ± 5.57 pg/mL). In addition, the secretion of IL-6 and IL-12 significantly decreased (*p* < 0.05) in *E. coli* with probiotics and in LPS with probiotics treatment when compared to only *E. coli*. and LPS, respectively. Also, the secretion of IL-12 significantly decreased (*p* < 0.05) in *S.* Typhi with probiotics treatment when compared to only *S.* Typhi. The secretion of IL-6 in *S.* Typhi with *L. paracasei* treatment showed no significant decrease (*p* > 0.05) when compared to only *S.* Typhi, while the secretion of IL-6 in *S.* Typhi with *B. breve* treatment showed no significant increase (*p* > 0.05) when compared to only *S.* Typhi.

One related study reported that before or after the LPS stimulus challenge, all probiotic supernatants significantly increased the amount of IL-10 secreted by monocyte-derived macrophages (MDM) [30]. IL-6 secretion is induced by *L. casei, L. lactis*, and *L. reuteri* CFS but it is not stimulated by *L. acidophilus* and *S. boulardii* [30]. Probiotic *E. faecium* OV3-6 suppressed the production of IL-6 and IL-12 but increased the production of IL-10 [73].

In summary, studying the anti-inflammatory activity of probiotics is essential for understanding their potential therapeutic applications in various health conditions. It sheds light on their mechanisms of action, guides clinical research, and contributes to the development of safe and effective interventions for managing inflammation-related disorders.

### 3.4. Gut Microbiota Using Feces as a Representation by Real-Time PCR

The human GI tract contains an abundant and diverse microbial community, consisting of more than 100 trillion microorganisms [75]. The major phyla of gut microbiota are Firmicutes (i.e., *Lactobacillus)*, Bacteroidetes (*Bacteroides* and *Prevotella)*, Actinobacteria (i.e., *Bifidobacterium*), Proteobacteria (i.e., *E. coli*), Fusobacteria, and Verrucomicrobia. Firmicutes and Bacteroidetes account for 90% of the gut microbiota [75]. The gut microbiota plays a critical role in host immunity and many diseases, and reflects the composition of the microbiome [76]. Studies have demonstrated spatial differences between the mucosal wall and lumen of the GI tract, as well as longitudinal gradients in the microbiota composition at various points along the GI tract. Therefore, feces are used as a proxy for most studies of intestinal microbiota and represent only a partial portion of microbial diversity in the colon [76]. Moreover, the study of the gut microbiome in feces is easier and more suitable than studying samples obtained via invasive biopsies and brushings during colonoscopies [76].

**Figure 3:**
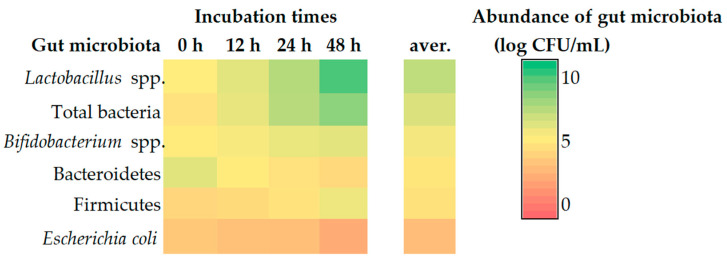
The abundance alteration heat map of gut microbiota, *Bifidobacterium* spp., *Lactobacillus* spp., Bacteroidetes, *Escherichia coli*, Firmicutes, and total bacteria in fermented feces at 0, 12, 24, and 48 h of incubation time.

The microbial standards of Firmicutes, Bacteroidetes, *Lactobacillus* spp., *Bifidobacterium* spp., *E. coli*, *Clostridium*, and total bacteria showed that the threshold cycle (C_t_) was approximately 30.13 to 11.16, 32.41 to 12.79, 24.91 to 9.28, 14.01 to 9.46, 30.32 to 10.54, 36.78 to 19.88, and 30.95 to 7.52, respectively. The correlation coefficient (R^2^) of all microbiological standard curves was greater than 0.99 (Supplementary Materials Figure S1), and the overall amplification efficiency was 129.44 ± 35.77%. The real-time PCR amplification of gut microbiota in the feces samples is shown in Figure S2 (Supplementary Materials). The abundance of *Bifibacterium* spp., *Lactobacillus* spp., Firmicutes, and total bacteria tended to increase, while the abundance of Bacteroidetes and *E. coli* tended to decrease from 0 to 48 h of feces fermentation times (Figure 3). The abundance of *Bifibacterium* spp. increased from 7.55 ± 0.55 CFU/g at 0 h to 7.81 ± 0.87, 7.97 ± 0.85, and 8.04 ± 0.98 CFU/g at 12, 24, and 48 h of incubation time, respectively. The abundance of *Lactobacillus* spp. increased from 7.66 ± 0.85 CFU/g at 0 h to 8.06 ± 0.56, 8.61 ± 0.58, and 9.06 ± 0.56 CFU/g at 12, 24, and 48 h of incubation time, respectively. The abundance of Bacteroidetes showed the highest decrease at 48 h of about 6.51 ± 0.36 CFU/g, followed by 24 and 12 h at about 6.91 ± 0.58 and 7.57 ± 0.02 CFU/g, respectively, when compared with 8.08 ± 0.67 CFU/g at 0 h of incubation time. Also, the abundance of *E. coli* exhibited the highest decrease at 48 h, followed by 24 and 12 h at about 3.85 ± 0.62, 4.98 ± 0.51, and 5.13 ± 1.02 CFU/g, respectively, when compared with 55.61 ± 0.49 CFU/g at 0 h of incubation time. The abundance of Firmicutes showed the highest increase at 48 h, approximately 7.93 ± 0.91 CFU/g, followed by 24 and 12 h at about 7.11 ± 0.58 and 6.56 ± 0.22 CFU/g, respectively, when compared with 0 h (6.28 ± 0.23 CFU/g) of incubation time. Moreover, the abundance of total bacteria showed the highest increase at 48 h, about 9.02 ± 0.31 CFU/g, followed by incubation times at 24 h, 8.58 ± 0.72 CFU/g, and at 12 h, which was 8.01 ± 1.02 CFU/g when compared with 0 h (7.06 ± 0.98 CFU/g). 

Probiotics (*L. paracasei* and *B. breve*) combined with prebiotics (INU, FOS, and GOS) have the potential to improve the gut microbiota in feces samples by increasing the abundance of *Lactobacillus* spp., *Bifidobacterium,* Firmicutes, and total bacteria in feces fermented for 48 h when compared with initial times. In addition, the abundance of Bacteroidetes and *E. coli* continued to decrease when using tested synbiotics. Similarly, the abundance of *Bifidobacterium* in the synbiotic group (*B. breve* MCC1274 and lactulose) in fermented human fecal communities showed significantly higher numbers when compared with the other treatment group [9]. In another study, synbiotics (short-chain FOS or FOS, each combined with one of four probiotics, *Lactobacillus fermentum* ME-3, *Lactobacillus plantarum* WCFS1, *Lactobacillus paracasei* 8700:2, or *Bifidobacterium longum* 46 increased bifidobacteria and the *Eubacterium rectale–Clostridium coccoides* group but decreased levels of *E. coli* [77]. Moreover, Saulnier et al. [77] concluded that modulating gut microbiota using the synbiotic strategy was more effective than with prebiotic or probiotic use alone. However, the total amount of bifidobacteria in cultures of infant fecal samples grew significantly more numerous when the GOS product was used, either by itself or combined with Beneo HP, with a large portion of this expansion being ascribed to the growth of *B. longum*. The total number of bifidobacteria and *B. longum* increased after using Beneo Synergy 1, while *E. coli* tended to decrease [35].

### 3.5. Organic Acid Contents

**Figure 4:**
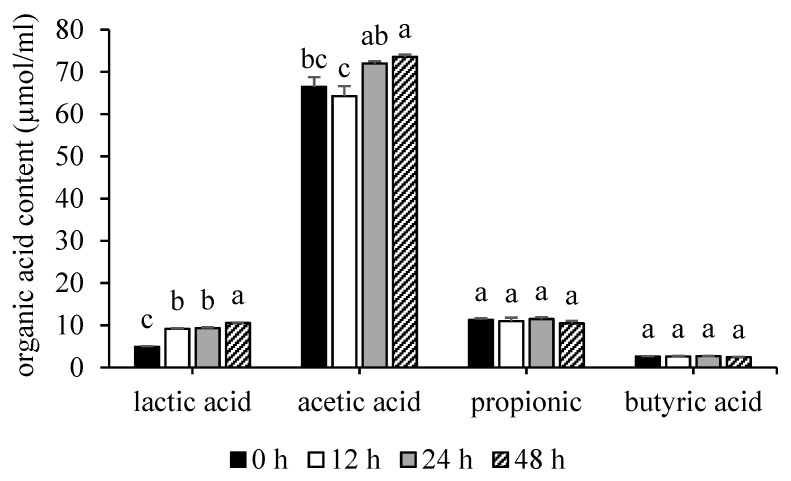
The organic acid contents, lactic, acetic, propionic, and butyric acid, in fermented feces at 0, 12, 24, and 48 h of incubation time. Significant differences (*p* < 0.05) were presented by different letters (a, b, and c) in the same type of organic acid.

The standards of lactic acid, acetic acid, propionic acid, and butyric acid had retention periods of 15.64, 17.72, 19.92, and 23.03 min, respectively. The coefficient of determination (R^2^) for lactic acid was 0.999, acetic acid was 0.999, propionic acid was 0.999, and butyric acid was 0.999, all of which met the acceptable limits (0.995 to 1.000). The results of organic acid contents are shown in Figure 4. The lactic acid content at 48 h (10.54 ± 0.10 µmol/mL) was significantly higher (*p* < 0.05) than at 24, 12, and 0 h (9.33 ± 0.18, 9.16 ± 0.11, and 4.89 ± 0.14 µmol/mL, respectively). The acetic acid content at 48 h (73.59 ± 0.50 µmol/mL) had significantly higher (*p* < 0.05) numbers than at 0 (66.47 ± 2.27 µmol/mL) and 12 h (64.27 ± 2.35 µmol/mL) but revealed no significantly difference at 24 h (71.99 ± 0.53 µmol/mL) of incubation time. Moreover, propionic acid concentrations at 0, 12, 24, and 48 h were 11.28 ± 0.38, 10.97 ± 0.84, 11.49 ± 0.41, and 10.47 ± 0.58 µmol/mL, respectively, and indicated no significant difference (*p* > 0.05) at each incubation time. Also, the butyric acid concentrations did not significantly differ (*p* > 0.05) at each incubation time, and at 0, 12, 24, and 48 h were 2.59 ± 0.12, 2.61 ± 0.12, 2.68 ± 0.05, and 2.47 ± 0.06, respectively.

Because SCFAs function as an important metabolite in the human body, their production by human gut microbiota is frequently employed as an indicator of gut health [33,78]. Acetic acid showed higher concentrations than other organic acids (Figure 4). The highest concentration of SCFAs was acetic acid, followed by propionic and butyric acid. Moreover, propionic and butyric acid showed no significance (*p* < 0.05) at any time of incubation. The most prevalent SCFAs in the human colon and feces (95%) are acetate, propionate, and butyrate, which are present in about a 60:20:20 molar ratio [33]. Thus, the reduced *E. coli* population may be directly related to the lower pH and higher acetic acid content [35]. In addition, the content of lactic acid indicated a trend to increase and was significantly highest (*p* > 0.05) at 48 h of incubation. Lactic acid can help reduce the pH of the digestive tract, which can improve the environment for beneficial bacteria and help in preventing the growth of infectious illnesses like *Salmonella* spp. or *E. coli* strains [79].

### 3.6. Stability Assessment of Synbiotics

The storage of synbiotics at 5 ± 3 °C and 30 ± 2 °C showed a significant decrease in the amount and survival rate of probiotics after storing for 6 months. The storage of synbiotics at 5 ± 3 °C for 1 month showed the significantly highest (*p* < 0.05) amount of probiotics, at about 7.71 ± 0.03 log CFU/mL, followed by storage at 3 and 6 months at about 7.57 ± 0.02 and 5.76 ± 0.02 log CFU/mL, respectively (Table 6). The amount of probiotics after storage at 30 ± 2 °C for 1, 3, and 6 months was 6.16 ± 0.02, 4.06 ± 0.02, and 3.49 ± 0.04 log CFU/mL, respectively. In addition, the moisture content of synbiotics after storing at 5 ± 3 °C at various times significantly increased (*p* < 0.05) from 5.85 ± 0.03% (0 month) to 5.97 ± 0.02% (6 months), while humidity at 30 ± 2 °C at every time significantly decreased (*p* < 0.05) from 5.86 ± 0.02% (1 month) to 4.99 ± 0.03% (6 months) (Table 6). The water solubility index of synbiotics after storing at 5 ± 3 °C showed the significantly highest amount (*p* < 0.05) at 0 month (85.11 ± 0.12%) and the lowest at 6 months (79.30 ± 0.31%). Also, storing at 30 ± 2 °C showed the significantly highest amount (*p* < 0.05) at 0 month (85.63 ± 0.14%) and the lowest at 6 months (80.26 ± 0.04%). The results suggested that for prolonged shelf-life, synbioitc products should be kept at 5 ± 3 °C, but if kept at 30 ± 2 °C they should not be stored for more than 1 month. Moreover, probiotic products with a long shelf life should be stored in vacuum sealed containers because oxygen concentration significantly impacts the survival and multiplication of probiotics during storage [80].

## 4. Conclusions

The use of microorganisms in food for humans must first be evaluated for safe and probiotic properties according to the main list of those currently used in *in vitro* tests for probiotic properties, including guidelines for evaluating probiotic properties in food. Both strains, *L. paracasei* and *B. breve*, exhibit potential probiotic properties and safety. Further, both probiotics possess cholesterol-lowering properties, which may decrease cholesterol in the blood. Therefore, *L. paracasei* and *B. breve* may be used as a starter culture or food additive for preparing fermented foods or food products. Moreover, synbiotics, a combination of probiotics, and a suitable ratio of mixed prebiotics, affect the growth of beneficial gut microbiota and decrease pathogens in feces fermentation. Positive changes in the quantity of metabolites such as lactic acid and SCFAs produced as a result of prebiotic fermentation may have beneficial effects on the health and functionality of the host’s GI tract, although additional *in vivo* research is necessary.

## Figures and Tables

**Table 1:** Real-time PCR primers for the quantification of gut microbiota.

Target Bacterial Group	Sequence (5′ → 3′)	Amplicon Length (bp)	Reference
*Bifidobacterium* spp.	F: TCGCGTCCGGTGTGAAAG	243	[38]
	R: CCACATCCAGCATCCAC		
*Lactobacillus* spp.	F: AGCAGTAGGGAATCTTCCA	345	[39]
	R: ATT(C/T)CACCGCTACACATG		
Bacteroidetes	F: GGA(A/G)CATGTGGTTTAATTCGATGAT	124	[40]
	R: AGCTGACGACAACCATGCAG		
Firmicutes	F: GGAG(C/T)ATGTGGTTTAATTCGAAGCA	127	[40]
	R: AGCTGACGACAACCATGCAC		
*Escherichia coli*	F: CATGCCGCGTGTATGAAGAA	400	[41]
	R: CGGGTAACGTCAATGAGCAAA		
Total bacteria	F: GTGCTGCATGGCTGTCGTCA	148	[42]
	R: ACGTCATCCCCACCTTCCTC		

**Table 2:** Antimicrobial activity.

Pathogenic Bacteria	Antimicrobial Activity			
	Agar Spot Test		Agar Well Diffusion	
	*L. paracasei*	*B. breve*	*L. paracasei*	*B. breve*
*Bacillus cereus* ATCC 11778	++	*++*	+	++
*Escherichia coli* ATCC 25922	+++	++	++	+++
*Pseudomonas aeruginosa* ATCC 27853	+++	+	++	++
*Salmonella enterica* subsp. *enterica* ser. Typhi DMST 22842	+++	++	+++	++
*Salmonella enterica* subsp. *enterica* ser. Typhimurium TISTR 1469	++	++	++	+
*Shigella sonnei* ATCC 25931	++	++	+++	++
*Staphylococcus aureus* ATCC 5923	+++	+++	++	+++

The diameter of inhibition zones of antimicrobial activity was determined: +, weak (inhibition zone < 3 mm); ++, moderate inhibition (inhibition zone 3–6 mm); +++, strong inhibition (inhibition zone > 6 mm); Remark: the inhibition zone does not include the diameter of the probiotic spot and well.

**Table 3:** Bile salt hydrolase (BSH) activity.

Conditions	BSH Activity					
	*L. paracasei*			*B. breve*		
	24 h	48 h	72 h	24 h	48 h	72 h
MRS + GCA	−	+	+	−	+	+
MRS + TCA	−	−	−	−	−	−
MRS + TDCA	−	+	+	−	+	+

GCA, glycocholic acid; TCA, taurocholic acid; TDCA, taurodeoxycholic acid; −, no BSH activity; +, shown BSH activity.

**Table 4:** Antibiotic susceptibility and antibiotic-resistant probiotics.

Pathogenic Bacteria	Concentrations (µg/disc)	Antibiotic Susceptibility	
		*L. paracasei*	*B. breve*
Ampicillin	10	S	S
Erythromycin	15	S	S
Gentamycin	10	S	S
Tetracycline	30	S	S
Vancomycin	30	R	R
Chloramphenicol	30	S	I
Kanamycin	30	R	I
Clindamycin	2	R	I
Streptomycin	10	S	S

S, susceptible (inhibition zone ≥ 20 mm); I, intermediate (inhibition zone 15–19 mm); R, resistant (inhibition zone < 14 mm).

**Table 5:** Secretion of cytokines by Caco-2 when treated with different treatments.

Treatments	Secretion of Cytokines (pg/mL)		
	IL-6	IL-10	IL-12
Untreated	5.85 ± 0.38 ^f^	7.81 ± 0.45 ^i^	0.53 ± 0.04 ^h^
LPS	55.15 ± 1.98 ^a^	83.93 ± 0.89 ^h^	7.56 ± 0.27 ^a^
*E. coli*	48.69 ± 1.19 ^b^	94.08 ± 2.00 ^g^	6.57 ± 0.28 ^b^
*S.* Typhi	43.60 ± 1.69 ^bc^	85.09 ± 1.45 ^h^	5.51 ± 0.15 ^c^
*L. paracasei*	17.85 ± 1.85 ^e^	264.65 ± 0.82 ^b^	2.71 ± 0.20 ^g^
*B. breve*	20.37 ± 0.94 ^e^	283.53 ± 4.10 ^a^	2.39 ± 0.21 ^g^
LPS + *L. paracasei*	43.66 ± 0.95 ^bc^	216.32 ± 5.57 ^c^	3.55 ± 0.07 ^f^
LPS + *B. breve*	45.69 ± 3.56 ^bc^	221.56 ± 3.09 ^c^	4.30 ± 0.04 ^e^
*E. coli* + *L. paracasei*	38.21 ± 1.77 ^d^	196.01 ± 2.39 ^e^	4.14 ± 0.07 ^e^
*E. coli* + *B. breve*	37.29 ± 1.99 ^d^	195.02 ± 1.19 ^ef^	4.40 ± 0.14 ^de^
*S.* Typhi + *L. paracasei*	41.15 ± 1.33 ^cd^	188.10 ± 2.00 ^f^	4.43 ± 0.18 ^de^
*S.* Typhi + *B. breve*	44.57 ± 1.61 ^bc^	208.41 ± 1.92 ^d^	4.87 ± 0.05 ^d^

The value of secretion of cytokines is shown as means ± SD of triplicate experiments. The different superscript letters represent a significant difference (*p* < 0.05) between the same column of cytokines. LPS is lipopolysaccharides.

**Table 6:** The survival rate, moisture content, and humidity of synbiotics during storage at various temperatures and months.

Month	Number of Survival Probiotics (log CFU/mL)		Moisture Content (%)		Water Solubility Index (%)	
	5 ± 3 °C	30 ± 2 °C	5 ± 3 °C	30 ± 2 °C	5 ± 3 °C	30 ± 2 °C
0	9.93 ± 0.01	9.91 ± 0.01	5.85 ± 0.03	5.86 ± 0.02	85.11 ± 0.07	85.17 ± 0.06
1	7.71 ± 0.03 *^,^**	6.16 ± 0.02 *^,^**	5.86 ± 0.02	5.83 ± 0.02	83.60 ± 0.26 *	84.41 ± 0.17 *
3	7.57 ± 0.02 *^,^**	4.06 ± 0.02 *^,^**	5.93 ± 0.02 *	5.14 ± 0.02 *	81.36 ± 0.07 *^,^**	83.41 ± 0.16 *^,^**
6	5.76 ± 0.02 *^,^**	3.49 ± 0.04 *^,^**	5.97 ± 0.02 *	4.99 ± 0.03 *	79.30 ± 0.31 *^,^**	80.26 ± 0.05 *^,^**

The values are represented as mean ± SD; * represents the values that had significant differences (*p* < 0.05) when compared with time 0 in the same row; and ** represents the values displayed significant differences (*p* < 0.05), compared between the temperature of each parameter in the same row.

## Data Availability

The data presented in the manuscript are available on request from the corresponding author.

## Supplementary Materials

The following are available online at https://www.mdpi.com/article/10.3390/foods12203847/s1, Figure S1: The standard curve of bacterial reference strains for real-time quantitative PCR: *Lactobacillus* spp. (**a**), *Bifidobacterium* spp. (**b**), Bacteroidetes (**c**), Firmicutes (**d**), *E. coli* (**e**), and total bacteria (**f**), Figure S2: The real-time PCR amplification of gut microbiota in the feces samples: *L. paracasei* (**a**), *B. breve* (**b**), Bacteroidetes (**c**), Firmicutes (**d**), *E. coli* (**e**), and total bacteria (**f**).
