# Supplementary figures and images for: Effects of Synbiotic Lacticaseibacillus paracasei, Bifidobacterium breve, and Prebiotics on the Growth Stimulation of Beneficial Gut Microbiota
Source: Foods. 2023 Oct 20;12(20):3847. doi: 10.3390/foods12203847 (PMC10606279; doi:10.3390/foods12203847)

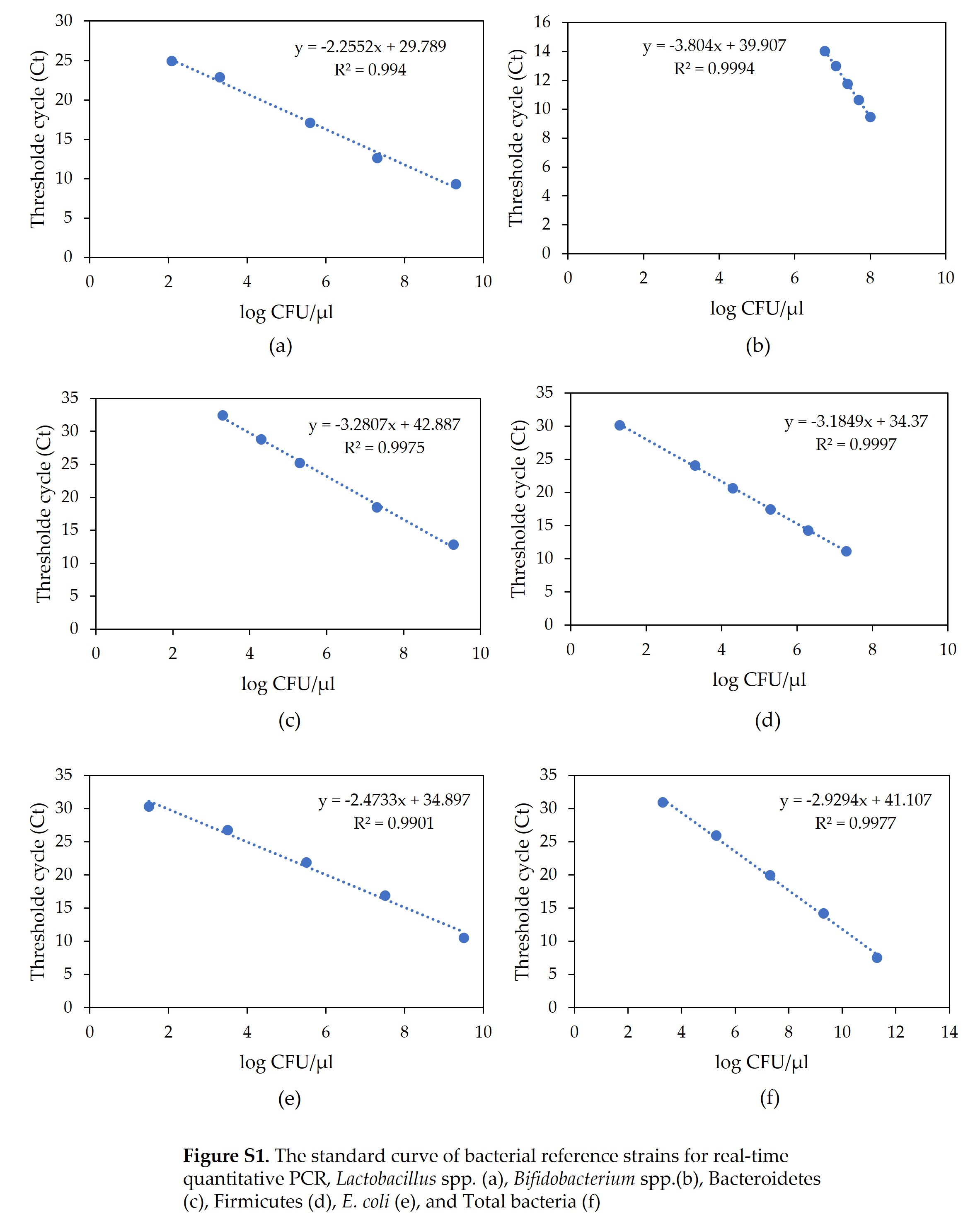

Supplement: Supplementary file 1 [file foods-12-03847-s001.zip › Figure S1.jpg]

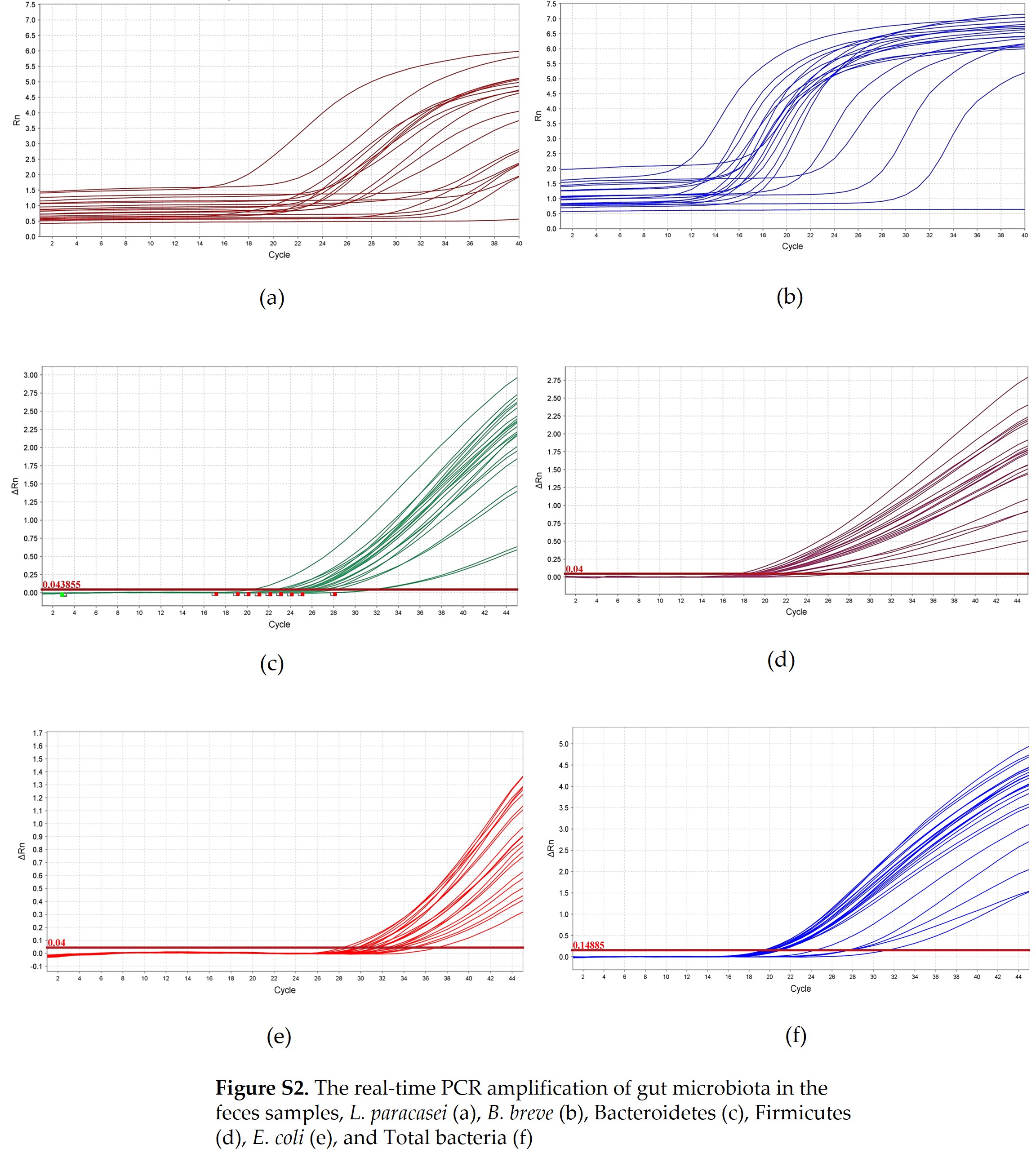

Supplement: Supplementary file 1 [file foods-12-03847-s001.zip › Figure S2.jpg]
